# Supplementary material for: Testing and healthcare seeking behavior preceding HIV diagnosis among migrant and non-migrant individuals living in the Netherlands: Directions for early-case finding
Source: PLoS One. 2022 Mar 4;17(3):e0264435. doi: 10.1371/journal.pone.0264435 (PMC8896686; doi:10.1371/journal.pone.0264435)
Supplement: S1 Appendix — (PDF) [file pone.0264435.s001.pdf]

aMASE Clinic Questionnaire

13.03.2013

DUTCH Version: GENERIC Final

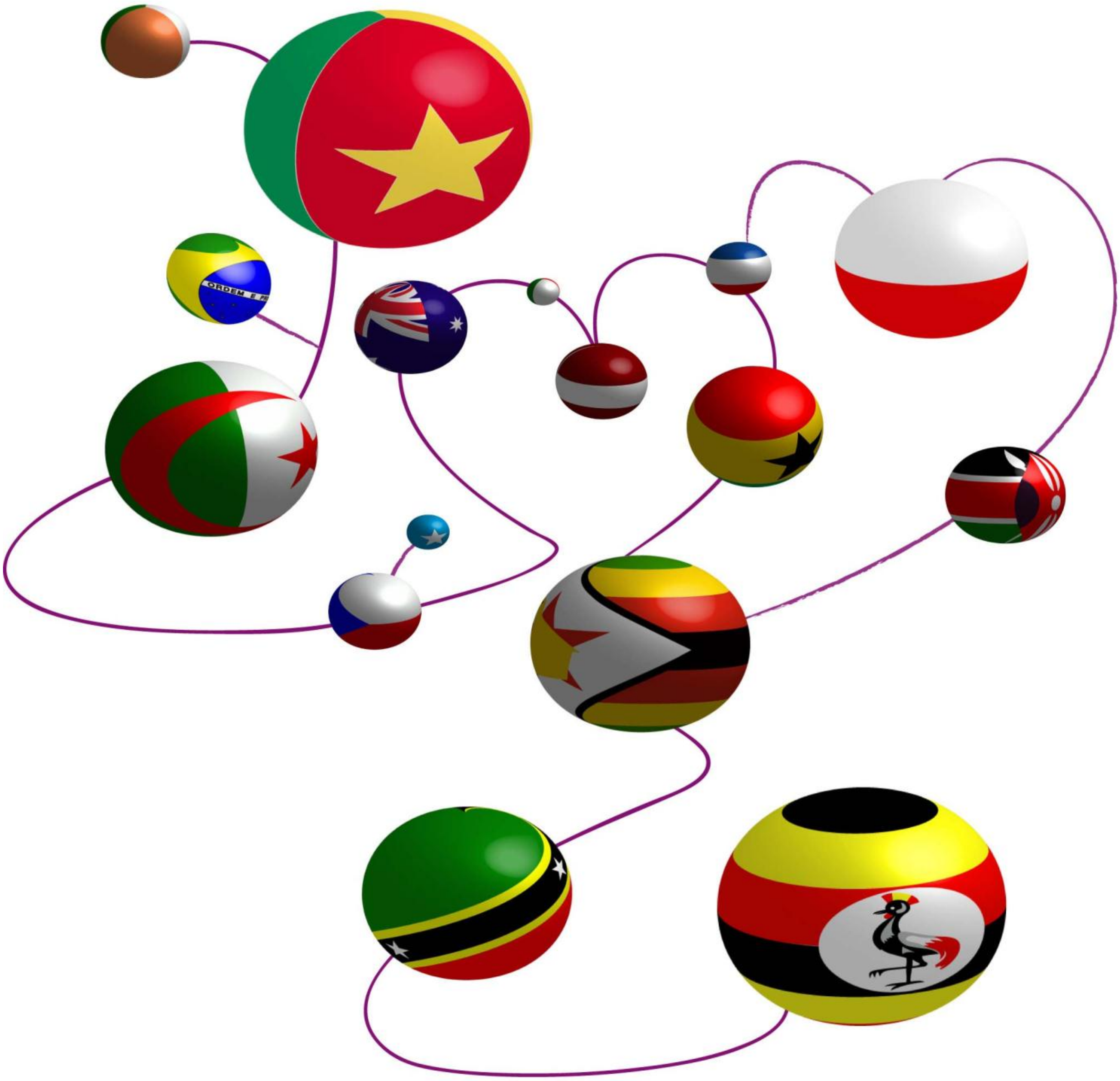

GENERAL REMARKS:

SC: Simple choice question

MC: Multiple choice question

©: Compulsory question

Notes and filters: remain in English (is only for the programmer)

Underlined in red: Please, check translation

QUESTIONNAIRE:

ID.date© **Datum van het interview**

Free text question in date format

NOTE
Date format DD/MM/YYYY. Default value=actual date. Check: if date is before actual date, error message.

ID.site© **Naam van de kliniek**

Free text question

NOTE
Restrict to three letters.

ID.language© **Taal die gebruikt is tijdens het interview**

SC

- ❑ *Engels*
- ❑ *Duits*
- ❑ *Spaans*
- ❑ *Amharic*
- ❑ *Nederlands*
- ❑ *Frans*
- ❑ *Grieks*
- ❑ *Italiaans*
- ❑ *Pools*
- ❑ *Portugees*
- ❑ *Russisch*
- ❑ *Turks*
- ❑ *Somalisch*
- ❑ *Arabisch*
- ❑ *Tigrinya*
- ❑

ID.code© **Voer het onderzoeksnummer in**

Free text question

NOTE
Restrict format to three letters and a three digit number

ID.interviewer© **Vult de participant de vragenlijst zelfstandig in?**

SC

- □ *Ja, de participant vult zelfstandig de vragenlijst in*
- □ *Nee, de participant krijgt hulp vanwege analfphabetisme, moeite hebben met het invullen via de computer of wegens een andere reden.*

Geef de computer aan de patiënt

(Text to the interviewed, layout in a separate page):

Bevorderen van de toegang tot gezondheidszorg voor migranten in Europa

Welkom

Geachte heer / mevrouw,
Hartelijk dank voor uw deelname in aMASE: het bevorderen van gezondheidszorg voor migranten in Europa. Dit project wordt gefinancierd door de Europese Unie om ons te helpen onze gezondheidszorg te ontwikkelen en deze meer relevant voor u te maken.

Sommige vragen in dit onderzoek zijn van persoonlijke aard; desalniettemin zijn uw antwoorden zeer nuttig. Deze enquête is strikt anoniem en vertrouwelijk. Schrijf niets op waardoor u geïdentificeerd zou kunnen worden.

Beantwoord de vragen zo volledig en precies mogelijk. Wanneer u vragen heeft, vraag het aan de onderzoeker of aan de receptie.
Met vriendelijke groeten,
Fiona Burns & Julia del Amo
EuroCoord, Work Package 14 leads.

0. © Ik bevestig dat ik het informatieblad (Versie 3.1: 19.10.2012) van aMASE (EuroCoord Work Package 14 study) gelezen en begrepen heb en dat ik de gelegenheid heb gekregen om vragen te stellen.

Ik begrijp dat mijn deelname vrijwillig is en dat ik het recht ben om me op ieder ogenblik terug te trekken, zonder opgave van reden en zonder consequenties van mijn wettelijke rechten.

SC
- ❑ ***Ik stem er in mee deel te nemen aan dit onderzoek*** ***⇨ Continue***
- ❑ *Ik wens niet deel te nemen aan dit onderzoek* ***⇨ End message #1***

1. © **In welk land vult u deze enquête in?**

SC

- ❑ *België*
- ❑ *Frankrijk*
- ❑ *Griekenland*
- ❑ *Duitsland*
- ❑ *Italië*
- ❑ *Nederland*
- ❑ *Portugal*
- ❑ *Spanje*
- ❑ *Zwitserland*
- ❑ *Verenigd Koninkrijk*

NOTES
1. Use answer as text subtitution referred to as {Q1}   *2. Use as filters referred to as BE, FR, GR, DE, IT, NL, PT, ES, CH and GB, respectively*

(Text to the interviewed): ***Dit eerste deel bevat algemene vragen over u en uw familie...***

2 © **Wat is uw situatie op dit moment?**

SC

- ❑ *Ik woon of verblijf 6 maanden of langer in {Q1}* ➩ ***Continue***
- ❑ *Ik woon of verblijf minder dan 6 maanden in {Q1}* ➩ ***End message #2***

3 © **Wat is uw leeftijd in jaren? (bij uw laatste verjaardag)**

Free text answer ➩ ***If <18 years go to End message #2***

NOTES
Restrict format to 2 digits

4 © **Bent u?**

SC

- ❑ *Vrouw*
- ❑ *Man*
- ❑ *Transgender man (geboren als man)*
- ❑ *Transgender vrouw (geboren als vrouw)*

5 © **In welk land bent u geboren? (gelieve een land te selecteren)**

DISPLAY LIST
Afghanistan (001)
Albanië (002)
Algerije (003)
Andorra (004)
Angola (005)
Antigua en Barbuda (006)
Argentinië (007)
Armenië (008)
Australië (009)
Oostenrijk (010)
Azerbeidzjan (011)
Bahamas, The (012)
Bahrein (013)
Bangladesh (014)
Barbados (015)
Wit-Rusland (016)

NOTES
1. Use answer as text subtitution referred to as {Q5}   *2. Number between brackets shows the code that will be assigned to that country, as order of answers will change depending on*

België (017)<br>Belize (018)<br>Benin (019)<br>Bhutan (020)<br>Bolivia (021)<br>Bosnië en Herzegovina (022)<br>Botswana (023)<br>Brazilië (024)<br>Brunei (025)<br>Bulgarije (026)<br>Burkina Faso (027)<br>Birma (028)<br>Burundi (029)<br>Cambodja (030)<br>Kameroen (031)<br>Canada (032)<br>Kaapverdië (033)<br>Centraal-Afrikaanse Republiek (034)<br>Chad (035)<br>Chili (036)<br>China (037)<br>Colombia (038)<br>Comoren (039)<br>Congo (Brazzaville) (040)<br>Congo (Kinshasa) (041)<br>Costa Rica (042)<br>Ivoorkust (043)<br>Kroatië (044)<br>Cuba (045)<br>Cyprus (046)<br>Tsjechië (047)<br>Denemarken (048)<br>Djibouti (049)<br>Dominica (050)<br>Dominicaanse Republiek (051)<br>Ecuador (052)<br>Egypte (053)<br>El Salvador (054)<br>Equatoriaal-Guinea (055)<br>Eritrea (056)<br>Estland (057)<br>Ethiopië (058)<br>Fiji (059)<br>Finland (060)<br>Frankrijk (061)<br>Gabon (062)<br>Gambia, The (063)<br>Georgia (064)<br>Duitsland (065)<br>Ghana (066)<br>Griekenland (067)<br>Grenada (068)<br>Guatemala (069)<br>Guinee (070)<br>Guinee-Bissau (071)<br>Guyana (072)	*the language used in the questionnaire: display in alphabetical order.*<br>*3. Check: if answer is similar to {Q1} go to ⇨ End message #2*

Haïti (073)
Heilige Stoel (074)
Honduras (075)
Hong Kong (076)
Hongarije (077)
IJsland (078)
India (079)
Indonesië (080)
Iran (081)
Irak (082)
Ierland (083)
Israël (084)
Italië (085)
Jamaica (086)
Japan (087)
Jordan (088)
Kazachstan (089)
Kenia (090)
Kiribati (091)
Korea, Noord (092)
Korea, Zuid (093)
Kosovo (094)
Koeweit (095)
Kirgizië (096)
Laos (097)
Letland (098)
Libanon (099)
Lesotho (100)
Liberia (101)
Libië (102)
Liechtenstein (103)
Litouwen (104)
Luxemburg (105)
Macau (106)
Macedonië (107)
Madagaskar (108)
Malawi (109)
Maleisië (110)
Malediven (111)
Mali (112)
Malta (113)
Marshall Eilanden (114)
Mauritanië (115)
Mauritius (116)
Mexico (117)
Micronesië (118)
Moldavië (119)
Monaco (120)
Mongolië (121)
Montenegro (122)
Marokko (123)
Mozambique (124)
Namibië (125)
Nauru (126)
Nepal (127)
Nederland (128)

Nederlandse Antillen (129)
Nieuw-Zeeland (130)
Nicaragua (131)
Niger (132)
Nigeria (133)
Noord-Korea (134)
Noorwegen (135)
Oman (136)
Pakistan (137)
Palau (138)
Palestijnse gebieden (139)
Panama (140)
Papoea-Nieuw-Guinea (141)
Paraguay (142)
Peru (143)
Filipijnen (144)
Polen (145)
Portugal (146)
Qatar (147)
Roemenië (148)
Rusland (149)
Rwanda (150)
Saint Kitts en Nevis (151)
Saint Lucia (152)
Saint Vincent en de Grenadines (153)
Samoa (154)
San Marino (155)
Sao Tome en Principe (156)
Saoedi-Arabië (157)
Senegal (158)
Servië (159)
Seychellen (160)
Sierra Leone (161)
Singapore (162)
Slowakije (163)
Slovenië (164)
Salomonseilanden (165)
Somalië (166)
South Africa (167)
Zuid-Korea (168)
Spanje (169)
Sri Lanka (170)
Soedan (171)
Suriname (172)
Swaziland (173)
Zweden (174)
Zwitserland (175)
Syrië (176)
Taiwan (177)
Tadzjikistan (178)
Tanzania (179)
Thailand (180)
Oost-Timor (181)
Togo (182)
Tonga (183)
Trinidad en Tobago (184)

Tunesië (185)
Turkije (186)
Turkmenistan (187)
Tuvalu (188)
Uganda (189)
Oekraïne (190)
Verenigde Arabische Emiraten (191)
Verenigd Koninkrijk (192)
Verenigde Staten van Amerika (193)
Uruguay (194)
Oezbekistan (195)
Vanuatu (196)
Venezuela (197)
Vietnam (198)
Jemen (199)
Zambia (200)
Zimbabwe (201)

6 © **In welk jaar verhuisde u voor het eerst naar {Q1}? (Gelieve vakanties en zakenreizen NIET mee te tellen)**
Free text answer

NOTES
1. Use answer as text subtitution referred to as {Q6}
2. Restrict format to 4 digits
Restrict answer from 1914 to 2013, both included

7© **Wanneer u terug denkt aan de zes maanden voorafgaand aan uw verhuizing naar {Q1}. In welk land woonde u toen?**
DISPLAY LIST (Similar list of countries)

8 © **Wat is uw nationaliteit?**
FREE TEXT

NOTES
FREE TEXT QUESTION (ONLY LETTERS). Provide two different boxes for those with double nationality.

9 **In welk dorp of stad woont u nu?**
Free text answer

NOTES
Force UPPERCASE

11 © **Hoe vaak bent u terug geweest naar {Q5} gedurende de AFGELOPEN 12 MAANDEN?**

SC
0
1
2
3
4
5
6
7
8
9

10 of meer

12© **Wat is de hoogste opleiding die u behaald heeft? Kies uit:**

SC
- ❑ *Minder dan lager onderwijs (basisschool/basis onderwijs)*
- ❑ *Lager onderwijs (basisschool/ basis onderwijs)"*
- ❑ *Middelbare school*
- ❑ *Een universitaire opleiding of gelijkwaardig*

12a ***Met welke leeftijd verliet u de middelbare school?***

Free text answer

FILTER	NOTES
ONLY IF Q12=3	*Restrict format to 2 digits*

12b © **Wat is de hoogste opleiding die u behaald heeft? Kies uit:**

SC
- ❑ Middelbaar Beroepsonderwijs (MBO)
- ❑ Hoger Beroepsonderwijs (HBO) of Universiteit

FILTER
Only IF COUNTRY=NETHERLANDS & Q 12=4

13 **Welke van deze omschrijvingen heeft betrekking op u? (Alles aanvinken wat van toepassing is)**

MC
Pop-up
- ❑ *Fulltime studie/school/training*
- ❑ *Fulltime werkend*
- ❑ *Parttime werkend*
- ❑ *Werkeloos en geregistreerd voor een uitkering*
- ❑ *Werkeloos en niet geregistreerd voor een uitkering*
- ❑ *Niet in staat om te werken (ziek op lange termijn of invaliditeit)*
- ❑ *Niet toegestaan om te werken (om immigratie redenen)*
- ❑ *Vrijwilligerswerk*
- ❑ *Thuis/familiezorg*
- ❑ *Gepensioneerd*

14 © **Bent u.... (alles aanvinken wat van toepassing is)**

SC
- ❑ *Getrouwd of in een wettelijk erkend geregistreerd partnerschap*
- ❑ *Vrijgezel en nooit getrouwd geweest*
- ❑ *Vrijgezel maar eerder getrouwd geweest*
- ❑ *Samenwonend met uw partner als een stel*
- ❑ *In een relatie maar niet samenwonend*

15 © **Woont uw vrouw/man/partner bij u in {Q1}?**

SC
- ❑ *Yes*
- ❑ *No*

FILTER
ONLY IF Q14=1

16 © **Heeft u kinderen?**

SC

- ❑ *Ja*
- ❑ *Nee*
- ❑ *Zeg ik liever niet*

17 **Voor hoeveel kinderen zorgt u op dit moment bij u thuis?**

SC

Pop-up

Free text answer

FILTER	NOTES
ONLY IF Q16=1	*Restrict format to 2 digits*

18© **Gaat u regelmatig naar een religieuze dienst?**

SC

- ❑ *Nooit*
- ❑ *Wekelijks of vaker*
- ❑ *Maandelijks of vaker*
- ❑ *Eén of twee keer per jaar*
- ❑ *Zeg ik liever niet*

19 **Welke religie?**

SC

Pop-up

- ❑ *Christelijk (Alle stromingen)*
- ❑ *Moslim/Islam*
- ❑ *Jodendom /Joods*
- ❑ *Boeddhisme/ Hindoeïsme / Sikhtempel*
- ❑ *Traditionele Afrikaanse religie*
- ❑ *Anders (Graag specificeren)*

FILTER
ONLY IF Q18=2,3,4

20 **Hoe zou u uw seksuele oriëntatie willen omschrijven?**

SC

Pop-up

- ❑ *Homo of lesbisch / Homoseksueel*
- ❑ *Heteroseksueel*
- ❑ *Biseksueel*
- ❑ *Anders (Graag specificeren)*

(Text to the interviewed): ***De vragen in dit onderdeel gaan over uw seksleven en uw seksuele gedrag.***
Noem bij het beantwoorden van deze vragen iedereen waarmee u ooit seks hebt gehad, ongeacht of het voor maar één keer of een paar keer was of met een reguliere partner of echtgenoot/echtgenote.

21 © **Heeft u seks gehad in de afgelopen 12 maanden?**

SC ❑ *Ja*
❑ *Nee*
❑ *Ik heb nooit seks gehad*

22a© Met hoeveel MANNEN heeft u seks gehad in de afgelopen 12 MAANDEN? (kies een waarde)

SC
❑ *Geen*
❑ *1*
❑ *2*
❑ *3-5*
❑ *6-10*
❑ *11-20*
❑ *21-50*
❑ *51-100*
❑ *101-499*
❑ *500 of meer*
❑ *Zeg ik liever niet*

FILTER	NOTES
ONLY IF Q21=1	*Q22a & Q22b SAME SCREEN SIDE BY SIDE*

22b© Met hoeveel VROUWEN heeft u seks gehad in de afgelopen 12 MAANDEN? (kies een waarde)

SC
❑ *Geen*
❑ *1*
❑ *2*
❑ *3-5*
❑ *6-10*
❑ *11-20*
❑ *21-50*
❑ *51-100*
❑ *101-499*
❑ *500 of meer*
❑ *Zeg ik liever niet*

FILTER	NOTES
ONLY IF Q21=1	*Q22a & Q22b SAME SCREEN SIDE BY SIDE*

23a© Met hoeveel MANNEN heeft u sinds uw aankomst in {Q1} seks gehad?

SC
❑ *Geen*
❑ *1*
❑ *2*
❑ *3-5*
❑ *6-10*
❑ *11-20*
❑ *21-50*
❑ *51-100*
❑ *101-500*
❑ *501-999*
❑ *1000 of meer*
❑ *Zeg ik liever niet*

FILTER	NOTES
ONLY IF Q21=1,2	*Q23a & Q23b SAME SCREEN SIDE BY SIDE*

23b© **Met hoeveel VROUWEN heeft u sinds uw aankomst in {Q1} seks gehad?**

SC

- ❑ *Geen*
- ❑ *1*
- ❑ *2*
- ❑ *3-5*
- ❑ *6-10*
- ❑ *11-20*
- ❑ *21-50*
- ❑ *51-100*
- ❑ *101-500*
- ❑ *501-999*
- ❑ *1000 of meer*
- ❑ *Zeg ik liever niet*

FILTER	NOTES
ONLY IF Q21=1,2	*Q23a & Q23b SAME SCREEN SIDE BY SIDE*

24a© **Met hoeveel MANNEN heeft u in totaal in uw leven seks gehad?**

SC

- ❑ *Geen*
- ❑ *1*
- ❑ *2*
- ❑ *3-5*
- ❑ *6-10*
- ❑ *11-20*
- ❑ *21-50*
- ❑ *51-100*
- ❑ *101-500*
- ❑ *501-999*
- ❑ *1000 of meer*
- ❑ *Zeg ik liever niet*

FILTER	NOTES
ONLY IF Q21=1,2	*Q24a & Q24b SAME SCREEN SIDE BY SIDE*

24b© **Met hoeveel VROUWEN heeft u in totaal in uw leven seks gehad?**

SC

- ❑ *Geen*
- ❑ *1*
- ❑ *2*
- ❑ *3-5*
- ❑ *6-10*
- ❑ *11-20*
- ❑ *21-50*
- ❑ *51-100*
- ❑ *101-500*
- ❑ *501-999*
- ❑ *1000 of meer*
- ❑ *Zeg ik liever niet*

FILTER	NOTES
ONLY IF Q21=1,2	*Q24a & Q24b SAME SCREEN SIDE BY SIDE*

25 © **Hoe vaak heeft u of uw seksuele partner een condoom gebruikt bij de seks sinds uw hiv diagnose?**

SC

- ❑ *Iedere keer*

FILTER
ONLY IF Q21=1,2

	❑ *Meestal*<br>❑ *Soms*<br>❑ *Nooit*<br>❑ *Zeg ik liever niet*	
26 ©<br><br>SC	**Heeft u of uw seksuele partner een condoom gebruikt bij de LAATSTE KEER dat u seks heeft gehad?**<br>❑ *Ja*<br>❑ *Nee*<br>❑ *Zeg ik liever niet*	**FILTER**<br>*ONLY IF Q21=1,2*
27<br><br>SC<br>Pop-up	**Wanneer u denkt aan de laatste persoon waar u seks mee heeft gehad, was deze**<br>❑ *Zeker hiv negatief (had geen hiv)*<br>❑ *Waarschijnlijk hiv negatief*<br>❑ *Niet zeker / Ik weet het niet*<br>❑ *Waarschijnlijk hiv positief*<br>❑ *Zeker hiv positief (had wel hiv)*	**FILTER**<br>*ONLY IF Q21=1,2*
28 ©<br><br>SC	**Heeft u ooit wel eens seks gehad met iemand in ruil voor geld, eten of drugs?**<br>❑ *Ja*<br>❑ *Nee*<br>❑ *Zeg ik liever niet*	**FILTER**<br>*ONLY IF Q21=1,2*
28a ©<br><br>MC	**In welk land heeft u seks gehad met iemand in ruil voor geld, eten of drugs ?(Vink alles aan wat van toepassing is)**<br>❑ *{Q1}*<br>❑ *{Q5}*<br>❑ *Ander land (Graag specificeren)*<br>❑ *Zeg ik liever niet*	**FILTER**<br>*ONLY IF Q28=1*
29 ©<br><br>SC	**Heeft u ooit geld betaald voor seks?**<br>❑ *Ja*<br>❑ *Nee*<br>❑ *Zeg ik liever niet*	**FILTER**<br>*ONLY IF Q21=1,2*
29a ©<br><br>MC	**In welk land heeft u geld betaald voor seks? (Vink alles aan wat van toepassing is)**<br>❑ *{Q1}*<br>❑ *{Q5}*<br>❑ *Ander land (Graag specificeren)*<br>❑ *Zeg ik liever niet*	**FILTER**<br>*ONLY IF Q29=1*

30 © **Heeft u ooit seks gehad tegen uw zin omdat iemand u bedreigde of fysiek geweld gebruikte (arm omdraaien, vasthouden, etc) om u zover te krijgen?**

SC
- ❑ *Ja*
- ❑ *Nee*
- ❑ *Zeg ik liever niet*

FILTER
ONLY IF Q21=1,2

(Text for the interviewed): ***De volgende reeks vragen gaat over het gebruik van drugs (zoals cannabis, heroïne, cocaïne, amfetaminen en steroïden). Niet van toepassing zijn medicijnen die zijn voorgeschreven door een arts.***

31 **Welke van de drugs hieronder heeft u in de afgelopen 5 jaar gebruikt? (Vink alles aan wat van toepassing is)**

MC
Pop-up
- ❑ *Ik heb geen enkele drugs gebruikt in de afgelopen 5 jaar*
- ❑ *Cannabis (marihuana, gras, hash, ganja, draw, skunk, weed, spliff)*
- ❑ *Amfetamines (speed, whizz, uppers, billy)*
- ❑ *Cocaïne of coke (charlie)*
- ❑ *Crack (rock, stones, white)*
- ❑ *Ecstasy (E, MDMA)*
- ❑ *Heroïne (smack, skag, H, brown, gear, horse)*
- ❑ *Acid, LSD (tabs, trips) of paddo's*
- ❑ *Ketamine (K, Special K)*
- ❑ *Crystal Meth (MethAmfetamine)*
- ❑ *GBL/GHB (G)*
- ❑ *Amyl Nitraten (poppers, vloeibaar goud, Rush)*
- ❑ *Andere niet voorgeschreven drugs*

NOTES
"I have not used any drugs in the last 5 years" is an exclusive answer

32 © **Heeft u ooit, ook al is het maar één keer, een naald gebruikt om een drug te injecteren? Het gaat NIET om dat wat u op doktersrecept hebt gebruikt.**

SC
- ❑ *Ja*
- ❑ *Nee*
- ❑ *Zeg ik liever niet*

33 **Wanneer heeft u voor het laatst een naald gebruikt om een drug te injecteren?**

SC
- ❑ *In de afgelopen 4 weken*
- ❑ *Tussen 4 weken en 1 jaar geleden*
- ❑ *Tussen 1 en 3 jaar geleden*
- ❑ *Langer dan 3 jaar geleden*

FILTER
ONLY IF Q32=1

34© **In welke landen heeft u een naald gebruikt om drugs of andere substanties te injecteren? (Vink alles aan wat van toepassing is)**

MC
- ❑ *{Q1}*

FILTER
ONLY IF Q32=1

- ❑ {Q5}
- ❑ *Ergens anders (Graag specificeren)*

35 **Heeft u ooit gebruik gemaakt van watjes, kookgerei of water waarvan u wist of dacht dat iemand anders deze al eerder had gebruikt?**

SC
- ❑ *Ja*
- ❑ *Nee*

FILTER
ONLY IF Q32=1

36 **Heeft u ooit een naald gebruikt om te injecteren waarvan u wist of dacht dat iemand anders deze al eerder had gebruikt?**

SC
- ❑ *Ja*
- ❑ *Nee*

FILTER
ONLY IF Q32=1

37 **Als u denkt aan de laatste keer dat u zich injecteerde met een naald in {Q1}, waar haalde u de naalden vandaan? (Vink alles aan wat van toepassing is)**

MC
- ❑ *Gekocht bij een apotheek of andere winkel*
- ❑ *Spuitomruil*
- ❑ *Outreach werk*
- ❑ *Vrienden / Andere mensen die drugs injecteren*
- ❑ *Gestolen*
- ❑ *Drugsdealer*
- ❑ *Anders (Graag specificeren)*

FILTER
ONLY IF Q34=1

(Text for the interviewed): ***Dit gedeelte gaat over hiv en AIDS…***

38 © In welk jaar is bij u hiv gediagnosticeerd?

SC *DISPLAY LIST*
- ❑ *2013*
- ❑ *2012*
- ❑ *2011*
- ❑ *2010*
- ❑ *2009*
- ❑ *2008*
- ❑ *2007*
- ❑ *2006*
- ❑ *2005*
- ❑ *2004*
- ❑ *2003*
- ❑ *2002*
- ❑ *Eerder*

NOTES
If diagnosis performed before 2008 → show a check window and if he is sure skip to end Thanks message #1

39 © **Waar bent u voor het EERST gediagnosticeerd?**

SC

- ❑ *{Q1}*
- ❑ *{Q5}*
- ❑ *Ergens anders (Graag specificeren)*

40d© **Bent u voor het EERST gediagnosticeerd in een..?**

SC

- ❑ *Verloskundigenpraktijk of bij de gynaecoloog (in relatie tot een zwangerschap)*
- ❑ *Ziekenhuis (bijv. op de spoedeisende hulp, tijdens een opname of bij een poliklinische afspraak))*
- ❑ *Huisartsenpraktijk (Huisarts, medisch huis)*
- ❑ *Kliniek voor seksuele gezondheid of hiv-testkliniek*
- ❑ *Centrum voor behandeling van drugverslaving / Methadonkliniek*
- ❑ *Gevangenis of detentiecentrum*
- ❑ *Gemeenschap / Outreach instelling (bv. buurthuis, mobiele faciliteit)*
- ❑ *Privé kliniek*
- ❑ *Anders (Graag specificeren)*

**FILTER**
ONLY IF Q39=1 & COUNTRY {Q1}= NL

40d_1 **Graag specificeren**

SC

- ❑ *Spoedeisende hulp in een ziekenhuis*
- ❑ *Opname in een ziekenhuis*
- ❑ *Ambulante afspraak*

**FILTER**
ONLY IF Q40d=2 & COUNTRY {Q1}=NL

41 **Waarom heeft u de hiv-test gedaan? (Vink alles aan wat van toepassing is)**

MC

- ❑ *Een arts heeft me geadviseerd om de test te doen vanwege gezondheidsproblemen*
- ❑ *Het was onderdeel van mijn routine gezondheidscheck*
- ❑ *Ik heb seks gehad met iemand waarvan ik wist/dacht dat deze hiv had*
- ❑ *Ik dacht dat ik risico liep*
- ❑ *Mijn kind was hiv-positief*
- ❑ *Het was onderdeel van mijn verzekerings- of hypotheekvoorwaarden*
- ❑ *Ik was zwanger*
- ❑ *Het was nodig voor mijn werk of sollicitatie*
- ❑ *Het was nodig voor het verkijgen van mijn visum*
- ❑ *Anders (Graag specificeren)*

42 © **Bent u ooit negatief getest op hiv (geen hiv) vóórdat hiv bij u werd vastgesteld?**

SC

- ❑ *Ja*
- ❑ *Nee*
- ❑ *Weet ik niet (meer)*

43 © **Waar heeft u uw laatste negatieve hiv-test gehad?**

SC

- ❑ *{Q1}*
- ❑ *{Q5}*
- ❑ *Ander land (Graag specificeren)*

FILTER
ONLY IF Q42=1

44 © **In welk jaar was uw laatste negatieve test? (bijv. 2001)**

SC

DISPLAY LIST

- ❑ *2013*
- ❑ *2012*
- ❑ *2011*
- ❑ *2010*
- ❑ *2009*
- ❑ *2008*
- ❑ *2007*
- ❑ *2006*
- ❑ *2005*
- ❑ *2004*
- ❑ *2003*
- ❑ *2002*
- ❑ *2001*
- ❑ *2000*
- ❑ *1999*
- ❑ *1998*
- ❑ *1997*
- ❑ *1996*
- ❑ *1995*
- ❑ *1994*
- ❑ *1993*
- ❑ *1992*
- ❑ *1991*
- ❑ *1990*
- ❑ *Eerder*

FILTER
ONLY IF Q42=1

45 **In welke landen heeft u seks gehad zonder condoom voordat u werd gediagnosticeerd? (Vink alles aan wat van toepassing is)**

MC

Pop-up

- ❑ *{Q1}*
- ❑ *{Q5}*
- ❑ *Ander land (Graag specificeren)*
- ❑ *Ik heb nog nooit seks gehad zonder condoom*

FILTER
ONLY IF Q21= (1 ; 2)

46 © **Heeft u uw reguliere/vaste partner verteld dat u hiv-positief bent?**

SC

- ❑ *Ja*
- ❑ *Nee*
- ❑ *Zeg ik liever niet*

FILTER
ONLY IF Q14= 1 ; 3 ; 4 ; 5)

47 **Wie heeft u verteld dat u hiv-positief bent? (Vink alles aan wat van toepassing is)**

MC
Pop-up

- ❑ *Ik heb nog nooit iemand verteld dat ik hiv-positief ben*
- ❑ *Mijn moeder / mijn vader*
- ❑ *Mijn zoon(s) of dochter(s)*
- ❑ *Een broer of zus*
- ❑ *Andere familieleden*
- ❑ *Mijn ex-partner*
- ❑ *Minimaal één goede vriend*

48 © Heeft u contact met goede doelen, verenigingen of maatschappelijke organisaties die ondersteuning bieden voor mensen die leven met hiv?

SC

- ❑ *Ja*
- ❑ *Nee*

49f Welke organisaties?

MC

- ❑ *HIV Vereniging Nederland ("HIV association Netherlands")*
- ❑ *Soa Aids Nederland (STI AIDS Netherlands)*
- ❑ *Aids fonds*
- ❑ *Poz and Proud*
- ❑ *Anders (Graag specificeren)*

**FILTER**
ONLY IF Q48=1 & COUNTRY {Q1}=NL

50a **Heeft u wel eens van PEP (Post Exposure Prophylaxis) gehoord, een anti-hiv medicijn dat gebruikt kan worden om overdracht van HIV te voorkomen na onbeschermde seks?**

SC

- ❑ *Ik heb nog nooit eerder van PEP gehoord*
- ❑ *Ik heb van PEP gehoord*
- ❑ *Ik heb gehoord van PEP en ik heb iemand aanbevolen om het te gebruiken*

**FILTER**
All EXCEPT CH

(Text for the interviewed): ***Deze vragen gaan over uw gebruik van gezondheidszorg in de TWEE JAAR voorafgaand aan uw hiv-diagnose...***

51d © Heeft u gebruik gemaakt van één van de volgende diensten in de TWEE jaar voorafgaand aan uw diagnose *{Q1}*?

MC

- ❑ *Opname in het ziekenhuis of andere instelling*
- ❑ *Een verloskundigenpraktijk of gynaecoloog (omdat u zwanger was)*
- ❑ *Eerste hulp / EHBO-afdeling*
- ❑ *Kliniek voor seksueel overdraagbare aandoeningen (soa)*
- ❑ *Een bezoek aan een polikliniek in het ziekenhuis*
- ❑ *Een abortuskliniek*

**FILTER**
ONLY IF Q39=1 & {Q1} =NL

- ❑ *Huisartsenpraktijk / Huisarts*
- ❑ *Tandarts*
- ❑ *Behandeling bij een psychiatrische instelling/Geestelijke gezondheidszorg*
- ❑ *Centrum voor behandeling van drugverslaving / Methadonkliniek*
- ❑ *Geen van het bovenstaande*

52© Heeft iemand het ooit over een hiv-test gehad met u tijdens deze bezoeken?

SC

- ❑ *Ja*
- ❑ *Nee*
- ❑ *Weet ik niet meer*

FILTER
ONLY IF Q51 (a/b/c/d) IS DIFFERENT FROM "NONE OF THE ABOVE"

53d Bij welke instanties heeft u over een hiv-test gesproken?

MC

- ❑ *Opname in het ziekenhuis of andere instelling*
- ❑ *Een verloskundigenpraktijkof gynaecoloog (in relatie tot een zwangerschap)*
- ❑ *Eerste hulp / EHBO-afdeling*
- ❑ *Kliniek voor seksueel overdraagbare aandoeningen (soa)*
- ❑ *Een bezoek aan een poli-kliniek in het ziekenhuis*
- ❑ *Een abortuskliniek*
- ❑ *Huisartsenpraktijk / Huisarts*
- ❑ *Tandarts*
- ❑ *Behandeling bij een psychiatrische instelling/Geestelijke gezondheidszorg*
- ❑ *Centrum voor behandeling van drugverslaving / Methadonkliniek*
- ❑ *Geen van het bovenstaande*

FILTER
ONLY IF Q52=1 & {Q1} =NL

(Text for the interviewed): ***De volgende vragen zullen gaan over andere infecties..***

54 © Heeft een dokter ooit de volgende infecties bij u vastgesteld voordat u met hiv werd gediagnosticeerd? (alles aanvinken wat van toepassing is)

MC

- ❑ *Hepatitis B*
- ❑ *Tuberculose*
- ❑ *Geen van beide*

54a In welk land bent u gediagnosticeerd met hepatitis B?(Vink alles aan wat van toepassing is)

MC

Pop-up

- ❑ *{Q1}*
- ❑ *{Q5}*

FILTER
ONLY IF Q54=1

❑ *Ander land (Graag specificeren)*

54b	**In welk land bent u gediagnosticeerd met tuberculose?(Vink alles aan wat van toepassing is)**	**FILTER**
MC<br>Pop-up	❑ *{Q1}*<br>❑ *{Q5}*<br>❑ *Ander land (Graag specificeren)*	*ONLY IF Q54=2*

55 ©	**Bent u ooit gediagnosticeerd met een andere seksueel overdraagbare aandoening (soa) dan hiv? (alles aanvinken wat van toepassing is)**	**FILTER**
MC	❑ *Nooit*<br>❑ *Ja, voor mijn hiv diagnose*<br>❑ *Ja, tegelijk met mijn hiv diagnose*<br>❑ *Ja, nadat ik met hiv was gediagnosticeerd*	ONLY IF Q21=(1 ;2)

55b	**In welk land bent u gediagnosticeerd met een andere soa (of soa's)? (alles aanvinken wat van toepassing is)**	**FILTER**
MC	❑ *{Q1}*<br>❑ *{Q5}*<br>❑ *Ander land (Graag specificeren)*	ONLY IF Q55 DIFFERENT FROM 1

56© **Bent u ooit gediagnosticeerd met hepatitis C? (alles aanvinken wat van toepassing is)**

MC ❑ *Nooit*

❑ *Ja, voor mijn hiv diagnose*

❑ *Ja, tegelijk met mijn hiv diagnose*

❑ *Ja, nadat ik met hiv was gediagnosticeerd*

56b	**In welk land bent u gediagnosticeerd met hepatitis C? (Vink alles aan wat van toepassing is)**	**FILTER**
MC	❑ *{Q1}*<br>❑ *{Q5}*<br>❑ *Ander land (Graag specificeren)*	ONLY IF Q56 DIFFERENT FROM 1

(Text for the interviewed): ***De volgende paar vragen gaan over de tijd tussen {Q6}, toen u voor het eerst verhuisde naar {Q1}, en VOORDAT u voor het EERST werd gediagnosticeerd met hiv***

57 © **Bent u ooit terug naar {Q5} gereisd in de tijd voor u met hiv gediagnosticeerd en uw aankomst in {Q1}?**

FILTER
ONLY IF Q39=1

SC ❑ *Ja*

Pop-up ❑ *Nee*

58 **Toen u terugreisde naar {Q5}, heeft u toen seks gehad gedurende deze periode? (Vink alles aan wat van toepassing is)**

FILTER
ONLY IF 57=1

MC
- ❑ *Ja, met mijn reguliere partner die in {Q5}woont*
- ❑ *Ja, met een nieuwe seksuele partner(s)*
- ❑ *Ja, met mijn reisgenoot*
- ❑ *Ja, met een vorige seksuele partner*
- ❑ *Ik heb geen seks gehad toen ik terug was in {Q5}*

(Text for the interviewed): ***Wanneer u denkt over uw huidige situatie...***

59 © **Neemt u momenteel hiv-medicijnen?**

SC ❑ *Ja*

❑ *Nee*

60 **In hoeverre bent u het eens of oneens met de volgende stellingen:**

FILTER
ONLY IF Q59=1

SC

	Helemaal mee eens	*Mee eens*	*Niet mee eens*	*Helemaal niet mee eens*
Ik volg de instructies van de arts met betrekking tot het nemen van mijn hiv-medicatie altijd op	❑	❑	❑	❑
Ik vind het moeilijk om regelmatig mijn hiv-medicatie te nemen	❑	❑	❑	❑

61 **Waarom neemt u momenteel geen hiv-medicatie? (Vink alles aan wat van toepassing is)**

FILTER
ONLY IF Q59=2

MC

Pop-up

- ❑ *Mijn arts zegt dat het nog niet nodig is*
- ❑ *hiv-behandeling is te duur en ik kan de kosten niet betalen*
- ❑ *Ik ben bang voor de bijwerkingen*
- ❑ *Ik wil het niet*
- ❑ *Ik ben bang dat andere mensen ontdekken dat ik hiv-positief ben*
- ❑ *Het is moeilijk om bij te houden met de vele verschillende tabletten*
- ❑ *Ik zit in een behandelingspauze in overleg met mijn arts*
- ❑ *Ik heb er geen toegang toe*
- ❑ *Anders (Graag specificeren)*

62d Wie betaalt er voor uw hiv-zorg (inclusief hiv-medicatie)?

SC

- ❑ *Overheid*
- ❑ *Ziektekostenverzekering*
- ❑ *Ik betaal zelf*
- ❑ *Een liefdadigheidsorganisatie*
- ❑ *Ik weet het niet*

FILTER
NL ONLY

63b © Heeft u een huisarts in {Q1}?

SC

- ❑ *Ja*
- ❑ *Nee*

FILTER
BE, DE, NL, PT, CH ONLY

64b **Heeft u geprobeerd om een huisartsenpraktijk te vinden in {Q1}?**

SC

- ❑ *Ja*
- ❑ *Nee*

FILTER
ONLY IF Q63b=2 & {Q1}= BE, DE, NL, PT, CH

65 **Weet uw huisarts in {Q1} dat u hiv-positief bent?**

SC

Pop-up

- ❑ *Ja*
- ❑ *Nee*
- ❑ *Ik weet het niet*

FILTER
IF Q63 (a OR b)=1 OR Q63d2=1 OR Q63d=1,2,3,4,5.

67a **Welke moeilijkheden heeft u gehad om gezondheidszorg te krijgen in {Q1} (Vink alles aan wat van toepassing is)**

MC

Pop-up

- ❑ *Geen moeilijkheden*
- ❑ *Ik kan geen huisarts vinden die mij als patiënt wil aannemen*
- ❑ *Ik weet nog steeds niet alles over mijn recht op medische zorg*
- ❑ *De openingstijden van de huisartskliniek zijn onhandig*
- ❑ *Er zijn lange wachttijden voor een afspraak in de huisartskliniek*
- ❑ *Er zijn lange wachttijden in de huisartskliniek*
- ❑ *Ik vertrouw de huisarts niet dat hij mijn gegevens geheim houdt*
- ❑ *Ik heb moeite met communiceren met het personeel van de huisartskliniek vanwege de taal*
- ❑ *Ik weet niet waar ik een huisarts kan vinden*
- ❑ *Anders (Graag specificeren)*

FILTER
BE, DE,NL, CH, GB ONLY

68 **Heeft u wel eens een afspraak gemist bij uw hiv-kliniek omdat u de reiskosten niet kon betalen (bijv. brandstof of bus/treinkaartje)?**

SC
- ❑ *Ja*
- ❑ *Nee*
- ❑ *Ik hoef geen reiskosten te betalen voor een bezoek aan de kliniek*

(Text for the interviewed): Als u denkt aan hiv medicatie en/of andere voorgeschreven medicatie, bijvoorbeeld bloeddrukverlagende medicijnen......

69 © **Neemt u wel eens te laat of geen medicatie omdat u de kosten voor het recept niet kunt betalen?**

SC
- ❑ *Ja*
- ❑ *Nee*
- ❑ *Ik hoef voor geen enkel medicijn te betalen*
- ❑ *Ik neem geen medicijnen*

(Text for the interviewed): *Wanneer u terug denkt aan de afgelopen 12 maanden....*

70f © **Het minimummaandloon in Nederland bedraagt €1,447 per maand (bruto). Als u dit vergelijkt, uw maandelijkse inkomen is....?**

SC
- ❑ *Veel meer dan dit*
- ❑ *Meer dan dit*
- ❑ *Ongeveer hetzelfde*
- ❑ *Minder dan dit*
- ❑ *Ik heb geen salaris*
- ❑ *Weet ik niet*
- ❑ *Zeg ik liever niet*

FILTER
NL ONLY

Q71 © **(Text for the interviewed): *Wanneer u terugdenkt aan de afgelopen VIER weken...***

SC

	Nooit	Zelden of soms	Vaak	Zeg ik liever niet
Heeft u ooit geen eten in huis gehad omdat u geen middelen had (bijv. geen geld, bonnen, etc)?	❑	❑	❑	❑
Ging u wel eens met een hongerig gevoel slapen omdat er niet genoeg te eten was?	❑	❑	❑	❑
Heeft u wel eens een hele dag en nacht niets gegeten omdat er niet genoeg te eten was?	❑	❑	❑	❑

72 **Bent u, sinds u in {Q1} bent, wel eens persoonlijk gediscrimineerd vanwege uw hiv-status?**

SC
- ❑ *Ja, maandelijks of vaak*
- ❑ *Ja, één of twee keer in het afgelopen jaar*
- ❑ *Ja, maar niet in het afgelopen jaar*
- ❑ *Nooit*

73 **Bent u, sinds u in {Q1} bent, wel eens vanwege uw ras, etniciteit of land van herkomst: gediscrimineerd, lastig gevallen, tegengehouden om iets te doen, of het gevoel gehad ondergeschikt te zijn?**

SC
- ❑ *Ja, maandelijks of vaak*
- ❑ *Ja, één of twee keer in het afgelopen jaar*
- ❑ *Ja, maar niet in het afgelopen jaar*
- ❑ *Nooit*

74 **Heeft iemand, sinds u in {Q1} bent, wel eens geprobeerd om u te discrimineren vanwege uw seksualiteit?**

SC
- ❑ *Ja, maandelijks of vaak*
- ❑ *Ja, één of twee keer in het afgelopen jaar*
- ❑ *Niet in het afgelopen jaar*
- ❑ *Nooit*

75b **Welke term beschrijft het best uw etnische achtergrond? (Kies er maar een)**

FILTER
BE, NL

SC
- ❑ *Afrikaans*
- ❑ *Amerikaans*
- ❑ *Aziatisch*
- ❑ *Europeaans*
- ❑ *Gemengd*
- ❑ *Arabier*
- ❑ *Midden-Oosten / Turks*
- ❑ *Roma*
- ❑ *Anders (graag specificeren)*

76 © **Wat omschrijft u het beste: (Vink alles aan wat van toepassing is)**

MC
- ❑ *Staatsburger van {Q1}*
- ❑ *EU-burger*
- ❑ *Permanente verblijfsvergunning voor {Q1}*
- ❑ *Tijdelijke verblijfsvergunning voor {Q1}*
- ❑ *Aanvraag voor verblijfsvergunning ingediend*
- ❑ *In {Q1} met een visum*
- ❑ *Asielzoeker*
- ❑ *Vluchtelingenstatus*
- ❑ *Studentenkaart /Visum*
- ❑ *Geen van het bovengenoemde*
- ❑ *Weet ik niet*

77 ***Hebt u aanmerkingen of suggesties met betrekking tot dit onderzoek?***

Free text answer

(Text for the interviewed):

Thanks message #1

Bedankt dat u de tijd hebt genomen om deze enquête in te vullen.
Klik op volgende

End message #1:

Bedankt dat u heeft overwogen om met dit onderzoek mee te doen. Als u nog vragen of opmerkingen heeft, dan kunt u contact opnemen met het onderzoeksteam.
Klik op volgende

End message #2:

Bedankt dat u de tijd heeft genomen om deel te nemen in dit onderzoek.
Dit onderzoek is alleen bedoeld voor mensen die ouder zijn dan 18 jaar, in de afgelopen 5 jaar met hiv zijn gediagnosticeerd en die langer dan 6 maanden in de opgegeven landen wonen.
Dank u voor uw tijd.
Klik op volgende

Druk op Verzenden
