# Supplementary material for: Testing and healthcare seeking behavior preceding HIV diagnosis among migrant and non-migrant individuals living in the Netherlands: Directions for early-case finding
Source: PLoS One. 2022 Mar 4;17(3):e0264435. doi: 10.1371/journal.pone.0264435 (PMC8896686; doi:10.1371/journal.pone.0264435)
Supplement: S2 Appendix — (PDF) [file pone.0264435.s002.pdf]

aMASE: Clinical data

Instructions:

Please follow these instructions to ensure that we retrieve a complete dataset.

1. Locate the respondents CLINIC number from the study logsheet.
2. Match the clinic number to the study number.
3. Locate the clinic notes.
4. Enter the data and upload.

If there are any difficulties please contact your local study coordinator.

ID.language © **Select a Language**

SC

- ❑ *Dutch*
- ❑ *English*
- ❑ *French*
- ❑ *German*
- ❑ *Greek*
- ❑ *Italian*
- ❑ *Portuguese*
- ❑ *Spanish*

NOTES
Questionnaire will be translated only into the countries' languages

CD1. © **Study Number**

Free text question

NOTES
Restrict format to three letters and a three digit number ***(Uppercase)***

CD2. © **Date of birth (DD/MM/YYYY)**

Free text question in date format

NOTES
Date format DD/MM/YYYY

CD3. © **Gender**

SC

- ❑ *Male*
- ❑ *Female*
- ❑ *Transgender Male*
- ❑ *Transgender Female*

CD4. © **Date of Diagnosis (MM/YYYY) If month not available please enter "01" for example: 01/2013**
Free text question in date format

NOTES
Date format ***MM/YYYY***

CD5. © **Pregnant at diagnosis**
SC
- ❑ *Yes*
- ❑ *No*
- ❑ *Not known*

FILTER
IF GENDER=female

CD6. © **Was there serological evidence of seroconversion?**
SC
- ❑ *Yes*
- ❑ *No*

CD7. © **Previous Negative HIV Test?**
SC
- ❑ *Yes*
- ❑ *No*
- ❑ *Not Known*

CD8. © **Date of previous negative test (MM/YYYY). If Day or Month not available please enter "01" for example: 01/2013**
Free text question in date format

FILTER	NOTES
If CD7=1	*Date format MM/YYYY*

CD9. © **ART ever initiated?**
SC
- ❑ *Yes*
- ❑ *No*
- ❑ *Not known*

CD10. © **Date ART Initiated (DD/MM/YYYY)**
Free text question in date format

FILTER	NOTES
If CD9=1	*Date format* DD/MM/YYYY

CD11. © **Currently on ART**
SC
- ❑ *Yes*
- ❑ *No*

FILTER
If CD9=1

CD4 COUNTS

(Text to the interviewed): ***CD4 Cell Counts (absolute cells/μ). If Day or Month not available please enter "01" for example: 01/05/2013***

CD12.		FILTER	NOTES
			All variables in the same table.
CD12a	CD4 count at diagnosis or (first available):		*Integer of 4 digits (max. 2000)*
CD12b	Date of CD4 Cell Count at diagnosis (or first available)		*Date format DD/MM/YYYY*
CD12c	CD4 count (absolute) cells/µl at 1st ART Initiation	*If CD9=1*	*Integer of 4 digits (max. 2000)*
CD12d	Date of CD4 Cell Count at 1st ART Initiation	*If CD9=1*	*Date format DD/MM/YYYY*
CD12e	Most recent CD4 count		*Integer of 4 digits (max. 2000)*
CD12f	Date of most recent CD4 cell count		*Date format DD/MM/YYYY*

CD12g **All CD4 counts completed or not known**
©
SC

- ❑ *Yes*
- ❑ *No*

Viral Load

(Text to the interviewed): ***Viral loads (copies/ml) If Day or Month not available please enter "01" for example: 01/05/2013***

CD13.		FILTER	NOTES
			All variables in the same table/page.
CD13a	Viral loads at diagnosis or (first available):		*Integer of 9 digits*
CD13b	Date of viral load at diagnosis (or first available)		*Date format DD/MM/YYYY*
CD13c	Viral load at 1st ART Initiation	*If CD9=1*	*Integer of 9 digits*
CD13d	Date of viral load 1st ART Initiation	*If CD9=1*	*Date format DD/MM/YYYY*
CD13e	Most recent viral load		*Integer of 9 digits*
CD13f	Date of most recent viral load		*Date format DD/MM/YYYY*

CD13g **All viral loads completed or not known**
©
SC

- ❑Yes
- ❑ No

CD14. © **Viral Type**

SC
- ❑ *HIV-1*
- ❑ *HIV-2*
- ❑ *Both HIV-1 and HIV-2*
- ❑ *Not Known*

CD15. © Viral Clade

MC

- ❑ *Not available*
- ❑ *A*
- ❑ *B*
- ❑ *C*
- ❑ *D*
- ❑ *E*
- ❑ *F*
- ❑ *G*
- ❑ *I*
- ❑ *H*
- ❑ *J*
- ❑ *K*
- ❑ *CRF01_AE*
- ❑ *CRF02_AG*
- ❑ *CRF03_AB*
- ❑ *Other CRFs*
- ❑ *URFs (unique recombinant form)*
- ❑ *N*
- ❑ *O*
- ❑ *P*

**FILTER**
Only if CD14=1 or CD14=3

CD16. © AIDS Defining Illnesses within three months of date of diagnosis?

SC
- ❑ *Yes*
- ❑ *No*
- ❑ *Not know*

CD17. © AIDS Defining Illness

MC

FILTER
Only if CD16=1

- ❑ *Recurrent Bacterial pneumonia*
- ❑ *Lymphoma*
- ❑ *Oeso. Candida*
- ❑ *PCP*
- ❑ *PML*
- ❑ *Cryptococccosis*
- ❑ *Mycobacteriosis*
- ❑ *CMV retinitis/encaphalitis*
- ❑ *Toxoplasmosis*
- ❑ *Tuberculosis, pulmonary*
- ❑ *Tuberculosis, disseminated/extrapulmonary*
- ❑ *Kaposi's Sarcoma, mucocutaneous/visceral*
- ❑ *HIV encephalopathy/ADC*
- ❑ *Other (specify)*

CD18. © Does the patient have Hepatitis B co-infection? (HbsAg+)

SC

- ❑ *Yes*
- ❑ *No*
- ❑ *Unknown*

CD19. © Does the patient have Hepatitis C co-infection? (PCR/bDNA)

SC

- ❑ *Yes*
- ❑ *No*
- ❑ *Unknown*

CD20. © Was the Hepatitis C infection acquired after the HIV diagnosis?

FILTER
Only if CD19=1

SC

- ❑ *Yes*
- ❑ *No*
- ❑ *Unknown*
